# 2268: A new framework for stakeholder engagement in early stage translational science

**DOI:** 10.1017/cts.2017.169

**Published:** 2018-05-10

**Authors:** Amy LeClair, Thomas Concannon, Virginia Kotzias, Allison Cole, Simona Kwon, Alexandra Lightfoot

**Affiliations:** Tufts University, Medford, MA, USA

## Abstract

OBJECTIVES/SPECIFIC AIMS: Stakeholder and community engagement (SCE) is a national priority for the National Center for Advancing Translational Science (NCATS). An established framework for stakeholder engagement exists for the latter stages (T2-T4) of translational, but no such framework currently exists for early stages of translational science (T1). Four Clinical and Translational Science Award (CTSA) hubs launched a collaboration to develop a new framework for engaging communities and stakeholders in T1 research. METHODS/STUDY POPULATION: We led structured individual and group discussions with T1 investigators to learn about: (1) the health decisions they seek to inform with research evidence, (2) the actors who make those decisions, and (3) the individuals and organizations that are affected by those decisions. In total, 18 individuals connected to 4 CTSA hubs participated in the discussions. Participants came from the fields ranging from basic chemistry and drug development to infectious disease and pediatrics and represented both methodological and topical experts. Focus groups lasted, on average, 1 hour, were audio recorded. Interviews lasted ~30 minutes. Audio recordings were transcribed and deidentified, and transcripts were coded using Dedoose™. We used a deductive-inductive procedure to develop the framework for stakeholder engagement in T1 research. A deductive codebook was development from the focus group and interview guides; emergent themes were added and the codebook was revised after preliminary inductive analysis. Two coders analyzed all transcripts using a constant comparison approach. We used an inductive process to identify themes and form them into a framework that could be used by T1 researchers in their work. The framework was developed through sequential reviews with coauthors and research participants. RESULTS/ANTICIPATED RESULTS: Preliminary findings suggest that stakeholders in early stage translational research (T1) do not fit into the same framework as those further down the translational spectrum (T2-T4). Basic scientists can identify stakeholders, however, and would like more guidance on who, how, and when to engage them in their research. DISCUSSION/SIGNIFICANCE OF IMPACT: By showing T1 researchers how to identify and involve their stakeholders in (1) defining research questions, (2) carrying out research activities, and (3) disseminating research evidence, this work has the potential to improve the use of basic science evidence in latter stages of translation from bench to bedside.

